# Conservation of the Mental Health and Care of the Insane

**Published:** 1914-10

**Authors:** 


### Conservation of the Mental Health and Care of the Insane.

The following is an abstract of an address made by Dr. Herring, secretary of the Commission in Lunacy of Maryland, delivered before the third annual meeting of the State Conference of Charities and Corrections:

"The conservation of the mental health of its people is one of the most important and far-reaching questions demanding the attention of a commonwealth. To paraphrase a familiar aphorism, 'Mental Health is Wealth,' so that a State which has adopted a well defined plan in regard to the mentally ill, embracing prevention, as well as provision of care and early treatment for those requiring it, is pursuing a wise, humanitarian, and economical policy.

Every government whether it be national or provincial, assumes in some manner the responsibility of caring for the mentally afflicted. Time does not permit, nor do I deem it essential on this occasion, to review the history of the care of the insane. The mentally deficient we have always had with us and always shall have. The question is, how best to provide for their care and treatment; and what steps are necessary to stem the ever increasing tide of insanity and feeble-mindedness. The Federal census of 1910 showed 187,454 persons in institutions for the insane in this country. At the present time the total number of insane and feeble-minded is in round numbers over 500,000. There are over 30,000 new cases of mental disease admitted to hospitals in the

United States each year, while the annual increase in the number of patients under treatment is about 6000. If all the States provided for their insane and feeble-minded as adequately as New York, Massachusetts, and Maryland, I venture to say there would be nearly 1,000,000 mental defectives in institutions. A concrete illustration of the prevalence of insanity is the fact that the number of hospital beds for the insane in New York City exceeds the number of hospital beds in all the general hospitals of that city. In Texas, fifty years ago, there were only sixty patients in a hospital for the insane. Today we have over 5000 known cases. The total number in the State is not known. The increase during the past fifty years has been nearly 100 cases a year.

In Maryland the increase is from 150 to 200 patients a year. This is largely because of the increased accommodations. There were over 2000 admissions of the insane and feeble-minded during 1913. The cost of caring for the insane in a State making adequate provisions exceeds any other single item of expense, even the amount expended for public education. The average cost of maintenance in institutions for the insane in the United States is about $175 per patient, making the total cost during the year 1910 for those in institutions $32,804,450. As it is estimated that the cost of the Panama Canal will be $325,201,000, and that the time for its completion will be about ten years, it is seen that the annual cost of caring for the insane is greater than the annual cost of the construction of the Panama Canal. The latter sum is so great that it was deemed necessary to distribute it over a number of years by the issuance of bonds; whereas, the cost of caring for the insane is an annual expense, which has to be met from the current revenues of the State. In order to state fairly the cost of mental diseases there must be added to this great sum the economic loss to the country through the withdrawal from productive labor of so many people in the prime of life. It has been stated by the United States Commissioner of Labor that the average value to the community of an adult between the ages of 18 and 45 is $700 a year. Estimated upon this basis, the annual economic loss to the United States through the confinement in institutions for the insane of 187,000 people is more than $130,000,000. If to this is added the cost of maintenance the total is more than $162,000,000, an amount equal to the entire value of the wheat, corn, tobacco, dairy products and beef products exported annually from the United States.

The care of the insane in the United States shows the widest variation, and it can be said that in most places it has failed to keep pace with the recent advances in the care of other classes of the sick. In many States a very large proportion of the insane patients receive only custodial care and many cases of curable mental disease fail to recover on this account. In other States many of the insane are in jails, and under such conditions neglect and cruelty are common. In only thirteen States are all the insane cared for in State hospitals. Almost all hospitals for the insane are overcrowded.

Political appointments are made in some institutions, and in certain States entire changes in hospital organizations take place with most States little or no scientific study of patients is made. There are no standards in such States for proper case records, or opportunities for medical research, and consequently the medical spirit is undeveloped.

In most localities patients during an early and very important period in their illness are cared for in jails, and are transferred to hospitals by policemen, or low salaried county officials. In many cases women are taken to hospitals by men. In some cases people with mental diseases have to remain in jails for weeks and even months before their cases can be taken up by the courts, and they can receive treatment in hospitals for the insane. The legal requirements for commitments are different in all States. In some States there is a jury system by which each person must be tried in open court, permitting family secrets to be revealed to idle lookers-on and become the source of gossip in the community, before admission to a hospital is possible. Under such conditions depressed patients, who would have been quite willing go to a hospital for treatment, if they had been permitted, often feel that they have been placed on trial for crime and develop delusions of sin and unworthiness which render their mental disease more serious and not infrequently injure their chances of recovery. Provisions for "emergency" commitment and voluntary commitments, the liberal use of paroles and provisions for after-care exist in a few States, but they are not employed in others and are quite unknown in some.

The trend of modern psychiatry is toward individualism rather than treating the disease per se. The patient is studied as a social unit. The environmental causes which contribute to his mental disorder are investigated, and before returning the patient to the community it is ascertained to what extent he can adapt himself to his new surroundings. This is embraced under what is known as the social service method, or, as it is sometimes termed, mental hygiene, which is a movement interested especially in the prevention of mental diseases.

### PREVENTION OF MENTAL DISEASE.

Humanitarianism and economic reasons alike call for organized efforts to control the spread of insanity. It is known that there are certain essential causes of mental disease and that some of these causes are within our control. Last year 449 persons died from typhoid fever in New York, but more than 500 persons with general paresis, all of whom, certain to die of their disease, were admitted during the same period and from the same population to hospitals for the insane; yet it is an amazing fact that even many of those most active in the field of venereal prophylaxis are not aware that this very prevalent and uniformly fatal disease depends on previous infections with syphilis. It is quite safe to say that 40 per cent of all first admissions to hospitals for the insane are preventable by means with which we are now familiar. This fruitful and most important field for work in the prevention of disease has thus far, very largely on account of lack of popular information, been practically neglected.

### POPULAR EDUCATION.

The principal reason for the abuses which are known to exist in the care of the insane is the amazing lack of general knowledge regarding mental diseases and the needs of those suffering from them. A preliminary survey of the field has shown that there are only fourteen States in which the care of the insane is under the supervision of a State board specifically charged with the duty alone. In most States the care of the insane depends upon boards performing many other functions, and in fourteen States there is no State board whatever which has any responsibility for the welfare of the insane. It seems plain, therefore, that it is an important part of the work of the National Committee for Mental Hygiene to stimulate popular interest in the welfare of a class of the sick which has been singularly neglected, and thus lay the ground work for the creation of local agencies capable of carrying on effective work for betterment.

### STATE CARE OF THE INSANE.

There are two methods of caring for the insane in this country, the county and the State system. With the former method in use, lack of progress, various abuses, and useless expenditures are always found. It is never desirable and should never be countenanced in any progressive State. It is not until the abuses existing under this system are brought plainly before the public that they can be corrected. The epoch-making reports of Dorothy Dix in a number of States, and especially the investigation of Willard in New York, led to the establishment of State control and care in New York, Maryland, Virginia, and other States. Under the county system the insane are confined in county almshouses and jails, illegitimate children are frequently born, abuses of all kinds exist, and as a result, the number of insane and feebleminded is constantly increasing. Under State control and care the picture is entirely different. The essential feature of State care is a central or State board of control. Such a board exists in every State where the insane are humanely and intelligently treated. The powers of such a board differs somewhat in various States, but the principles are the same. The system advocated now is for comparatively small hospitals, which shall minister to a district in the State. Inexpensive cottages grouped around a central administration group is the plan followed by all modern institutions. A careful survey and study of the conditions in the State should be made by the board of control, and a comprehensive plan outlined which will be elastic and adapted to all future requirements.

Instead of building large hospitals with their pseudo-economy we must aim to give well defined districts what they need, not only to mend but to prevent a gradual increase of burdens. A sufficient number of small, live, active hospitals, from which work can be done on the spot, and according to the needs of the locality, means infinitely more than a few huge asylums and monuments of resignation, no matter what excellent work is done within them. As long as they fail to reach out, our hospitals for the insane are standing approvals of fatalism concerning faulty ways of life."

---

At the recent meeting of the State Pharmaceutical Association, held in Dallas, the druggists of the State went on record as favoring more stringent laws regarding the sale of narcotic drugs. They passed a resolution which will ask that a law be passed making it an offense for any druggist in Texas to sell narcotic and injurious drugs to any other than a registered and licensed physician or directly to a veterinary or dentist. The resolution will further provide that the last two named must show for what purpose they are purchasing the drugs. A heavy penalty is to be asked for violations of the law.

